# Population biology of malaria within the mosquito: density-dependent processes and potential implications for transmission-blocking interventions

**DOI:** 10.1186/1475-2875-9-311

**Published:** 2010-11-04

**Authors:** Thomas S Churcher, Emma J Dawes, Robert E Sinden, George K Christophides, Jacob C Koella, María-Gloria Basáñez

**Affiliations:** 1 Department of Infectious Disease Epidemiology, School of Public Health, Faculty of Medicine, Imperial College London, UK; 2 Division of Cell and Molecular Biology, Faculty of Life Sciences, Imperial College London, UK; 3 Division of Biology, Faculty of Natural Sciences, Imperial College London, UK

## Abstract

**Background:**

The combined effects of multiple density-dependent, regulatory processes may have an important impact on the growth and stability of a population. In a malaria model system, it has been shown that the progression of *Plasmodium berghei *through *Anopheles stephensi *and the survival of the mosquito both depend non-linearly on parasite density. These processes regulating the development of the malaria parasite within the mosquito may influence the success of transmission-blocking interventions (TBIs) currently under development.

**Methods:**

An individual-based stochastic mathematical model is used to investigate the combined impact of these multiple regulatory processes and examine how TBIs, which target different parasite life-stages within the mosquito, may influence overall parasite transmission.

**Results:**

The best parasite molecular targets will vary between different epidemiological settings. Interventions that reduce ookinete density beneath a threshold level are likely to have auxiliary benefits, as transmission would be further reduced by density-dependent processes that restrict sporogonic development at low parasite densities. TBIs which reduce parasite density but fail to clear the parasite could cause a modest increase in transmission by increasing the number of infectious bites made by a mosquito during its lifetime whilst failing to sufficiently reduce its infectivity. Interventions with a higher variance in efficacy will therefore tend to cause a greater reduction in overall transmission than a TBI with a more uniform effectiveness. Care should be taken when interpreting these results as parasite intensity values in natural parasite-vector combinations of human malaria are likely to be significantly lower than those in this model system.

**Conclusions:**

A greater understanding of the development of the malaria parasite within the mosquito is required to fully evaluate the impact of TBIs. If parasite-induced vector mortality influenced the population dynamics of *Plasmodium *species infecting humans in malaria endemic regions, it would be important to quantify the variability and duration of TBI efficacy to ensure that community benefits of control measures are not overestimated.

## Background

Density-dependent processes that regulate population growth are common in host-parasite systems and can influence the resilience of an infection to control interventions [1]. In a model malaria-mosquito system, the progression of *Plasmodium berghei *through *Anopheles stephensi *depends non-linearly on parasite density [2]. Two types of density dependence operate during sporogony in this system. Firstly, the transitions from the female (macro-)gametocyte to ookinete, ookinete to oocyst, and oocyst to sporozoites are all restricted at high parasite densities. These negative density-dependent processes limit sporogony at high parasite densities (when the per parasite rate of transition to the next stage tends to zero), but are relaxed as density decreases. Secondly, an additional, positive density dependence impedes the transformation from ookinete to oocyst in mosquitoes with a low number of ookinetes [2]. This mechanism (the Allee effect) initially facilitates transmission as ookinete density increases, but will make it unstable at low parasite densities.

In addition to this density-dependent sporogonic development, the mosquito's (and thus parasite's) survival could be influenced by parasite density. Indeed, there is growing evidence that the mosquitoes survival depends on its age [3,4] and that *Plasmodium *may influence the life-expectancy of the mosquito [5] (although a meta-analysis of laboratory experiments investigating the effect of the malaria parasite on mosquito survival yielded inconclusive results [6]). In these experiments, the mortality rate of *An. stephensi *was dependent on its age and on the presence and density of *P. berghei *[7]. These density-dependent processes, identified in the *P. berghei-An. stephensi *model system, may operate in other parasite-vector combinations [8,9], including those relevant to human malaria [10-14]. Therefore, the interactions between these different positive and negative density-dependent processes may have important implications for the control of human malaria.

The intensity of malaria transmission in endemic areas is typically measured by the entomological inoculation rate (EIR); the annual number of infectious bites received by a person living in such areas. This metric makes the assumption that the infectiousness of a mosquito is the same irrespective of how many sporozoites are within the salivary glands. However, recent evidence indicates that there is a correlation between the number of *P. berghei *sporozoites at the bite site and the probability that the mouse host will go on to develop blood infection [15]. The importance of parasite density as a determinant of the potential for malaria transmission from the vector to the human population would depend on whether there is a correlation between the number of sporozoites within the salivary glands, the number injected into the human host, and ultimately the probability of acquiring the infection. Using quantitative PCR, Medica & Sinnis [16] recently showed that there was a statistically significant and positive correlation in the *Plasmodium yoelii-An. stephensi *system, despite substantial variability in the number of sporozoites injected between different mosquitoes and over time from the same mosquito. Notwithstanding such a correlation, the presence of parasite-induced, density-dependent vector mortality would make parasite density important so both sporozoite presence and density shall be presented in this paper.

Density-dependent parasite growth may impact upon various malaria control strategies. A number of promising control measures are being developed with the goal of reducing the incidence of human malaria by blocking transmission to and from the mosquito vectors. These include transmission-blocking vaccines (TBVs), which target antigens expressed on the malaria parasite within the mosquito [17], biological agents to prime the mosquitoes' immune system to reduce its infectivity [18], and the use of refractory, genetically modified mosquitoes to reduce infection of the mosquito [19]. However, most of these transmission-blocking interventions (TBIs) are, at present, only partially effective, so it is important to understand how their interaction with the non-linear processes taking place in the vector would influence overall transmission. Potential vaccines under investigation aim to target different *Plasmodium *life-stages within the vector [20]. Understanding how the population dynamics of the parasite within the mosquito may enhance or hinder the impact of a TBI could guide decisions as to which stage(s) to prioritize as target(s).

In this paper, mathematical models are used to investigate the potential effect on malaria transmission of the multiple density-dependent processes that have been identified in the *P. berghei-An. stephensi *model system. Since deterministic models can underestimate the cumulative effect of multiple non-linear functions, an individual-based stochastic model is used to capture the changes in parasite density over the different stages of sporogonic development. The highly overdispersed distribution of parasites recorded among mosquitoes [2,16,21-23] is also modelled explicitly, as parasite aggregation will, on average, increase the influence of density-dependent regulatory processes [24]. The results of the model are used to re-fit the mortality data from mosquito experimental infections reported by Dawes *et al *[7] to investigate how different parasite life-stages may influence vector mortality (in [7] it was assumed that the ookinete stage was responsible for excess mosquito mortality). The full model is then used to investigate how interventions targeting different within-vector parasite life-stages would influence the transmission dynamics of *Plasmodium*.

## Methods

**Figure 1:**
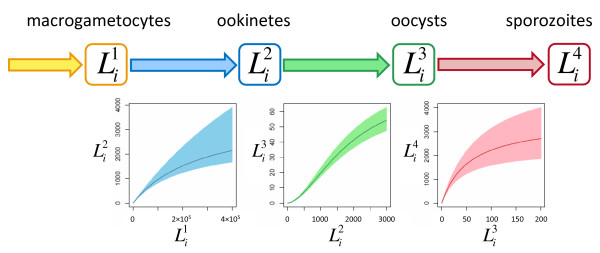
**Graphical description of the mathematical model describing sporogonic development within the mosquito prior to the introduction of an intervention**. The model charts the number of macrogametocytes, $L_{i}^{1}$, ookinetes, $L_{i}^{2}$, oocysts, $L_{i}^{3}$, and salivary gland sporozoites, $L_{i}^{4}$, within mosquito *i*. For a full list of notation see Additional file 1. The graphs under each arrow indicate the density-dependent processes describing the number of parasites which develop into the subsequent life-stage. Shaded areas indicate the 95% confidence intervals for the best-fit model fitted in [2].

A stochastic individual-based model was used to calculate the number of macrogametocytes, ookinetes, oocysts and salivary gland sporozoites at different times post-feeding in every mosquito of the hypothetical population. To facilitate comparison with published data the same post-feeding times are used as in [2], as these represent typical time-points at which mosquitoes are dissected to assess sporogonic development. Let $L_{i}^{j}$ indicate the number of parasites of life-stage *j *within mosquito *i*, be it macrogametocytes ingested ($L_{i}^{1}$) at cessation of feeding, ookinetes at 15 hours ($L_{i}^{2}$), oocysts at day 10 ($L_{i}^{3}$), or sporozoites in the salivary glands at day 21 ($L_{i}^{4}$). The life-expectancy of each mosquito was then estimated from data in Dawes *et al *[7] and used to quantify the contribution of each mosquito to overall transmission. A graphical representation of the model and the different density-dependent functions describing sporogonic development are given in Figure 1. A full description of the model and parameter values can be found in Additional file 1.

### Mosquito life-expectancy

It is unknown which stage(s) of *Plasmodium *development cause(s) the excess mortality in the mosquito. Ookinetes could increase susceptibility to bacterial infection as they perforate the mosquito midgut [25], whilst all three stages, ookinetes, oocysts, and sporozoites, could cause physiological disruption [26], nutrient depletion [26,27] or costly immune responses [28]. Assessing the parasite density in dead mosquitoes can be difficult (unless a mosquito is dissected shortly after its death, parasite counts are unreliable), so studies investigating parasite-induced vector mortality typically explore the association between mosquito mortality and the estimated number of parasites ingested. If later sporogonic stages cause mosquito mortality, any statistical investigation must take into consideration the highly non-linear relationship between the number of parasites ingested and the number developing into the stage causing the mortality. Otherwise parasite-induced vector mortality would be harder to detect. The relative success of different TBIs will be influenced by which sporogonic stage(s) cause(s) vector mortality. Therefore, scenarios are investigated in which vector mortality is associated with ookinete load; oocyst burden at day 10, or number of salivary gland sporozoites at day 21 post-feeding by re-fitting the empirical (parabolic) hazard function of [7] to the mosquito mortality rate obtained by these authors varying the life-stage that is responsible for the excess mortality. The Akaike information criterion (AIC) was used to distinguish between model fits (the best supported model being the one with the lowest AIC) [29].

### Variability in intervention efficacy

If vector mortality were determined by parasite load and not just parasite presence or absence, any variability in intervention efficacy would influence its overall effectiveness. Vaccinated populations typically generate a wide range of antibody responses [30] and the efficacy of other interventions may also vary. A partially efficacious intervention, which reduces parasite density but fails to clear infection, may increase the life-expectancy of a mosquito without substantially reducing its infectivity. This could lead to the intervention actually increasing rather than decreasing overall transmission.

By way of illustration, the variability in efficacy of all the interventions investigated in this paper was modelled on the range of responses expected from a single immunogenic TBV. Though the distribution of antibody titres generated by any TBV is not well known, it is expected to be similar to that in other human vaccines, which tend to show a log-normal distribution of antibody concentrations [30,31]. The relationship between antibody concentration and TBV efficacy is typically highly non-linear (hyperbolic) and can be described using the Hill equation [31] (see Protocol S1). This generates a skewed distribution of intervention efficacies whose mean is always closer to 50% than the median (i.e. a negative skew at high mean efficacies and a positive skew at low mean efficacies). For comparability it was assumed that all interventions reduce the production of the specific life-stage at which they are targeted, and not additionally the production of a subsequent stage. This is analogous to TBV antibodies attacking surface proteins of a particular life-stage as soon as they appear.

### Lifetime mosquito contribution to transmission

To aid interpretation of the complex concepts investigated in the paper, a measurement of parasite transmission is devised based on the contribution of an individual mosquito to onwards transmission over its lifetime (assuming that infection is acquired during its first bloodmeal). This method of estimating the lifetime transmission potential of a mosquito has been used in other mathematical approaches, though the exact definitions vary [32,33]. In this paper, the definition will depend on whether it is assumed that the potential for malaria transmission from vectors to humans is determined by the presence or the density of salivary gland sporozoites. For the former, onwards transmission is defined as "the mean number of infectious bites made per mosquito during its lifetime". For the latter, onwards transmission is "the mean number of salivary gland sporozoites available to be injected per mosquito during its lifetime". These metrics allow transmission to be compared between mosquitoes that ingest different gametocyte densities, irrespective of other contributing factors such as the mosquito to human ratio, the human blood index, and the endemicity of malaria.

## Results

### Cumulative impact of density-dependent sporogonic development

**Figure 2:**
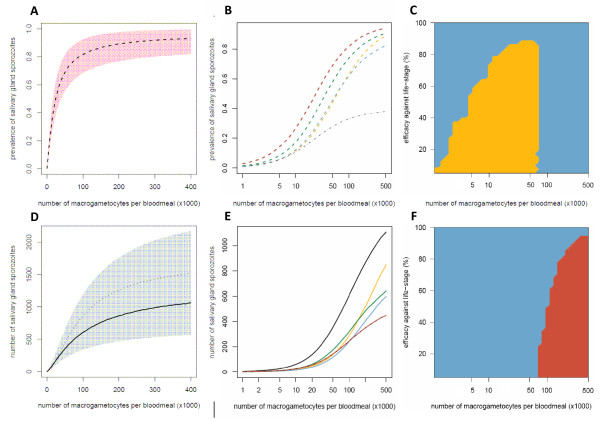
**The impact of transmission-blocking interventions which target different parasite life-stages on the prevalence and density of salivary gland sporozoites**. The Figure shows the relationship between the number of macrogametocytes ingested and the mean prevalence of infectious mosquitoes (dashed lines, A and B) or the mean number of salivary gland sporozoites per mosquito (solid lines, D and E). The model (described in Protocol S1) was run with either no intervention (black line) or representing an intervention which reduced the production of a *Plasmodium *life-stage by 60%: macrogametocytes (yellow line); ookinetes (blue line); oocysts (green line); salivary gland sporozoites (red line). The grey dotted-dashed line in panel B and panel E (where it lies on top of the red line) indicates an overall reduction in sporozoite density/prevalence of 60% as a benchmark for comparison. The shaded areas of panels A and D depict the 95% confidence intervals for the best-fit model. Panel D illustrates the importance of using an individual-based model (with which to account for parasite aggregation), as simply combining the three density-dependent functions within a mean-based, deterministic model (thin dotted-dashed line) underestimates the severity of the non-linear relationship. Panels C and F show which is the best life-stage to target to reduce transmission for a range of gametocyte densities and intervention efficacies: macrogametocytes (yellow surface); ookinetes (blue surface); sporozoites (red surface).

The cumulative impact of the multiple density-dependent processes acting on the progression of *P. berghei *through the mosquito makes the relationship between the number of macrogametocytes ingested and the presence or number of salivary gland sporozoites highly non-linear (Figures 2A, D). In highly infected vertebrate hosts, reducing the number of macrogametocytes ingested by 50%, from 300,000 to 150,000, reduces sporozoite prevalence by only ~6% and sporozoite density by ~24%. The number of vertebrate hosts with very high gametocytaemia is likely to be relatively small as the majority of infected individuals tend to have intermediate or low gametocyte densities (see [34], though care should be taken when comparing gametocyte densities between species as *P. falciparum *typically has higher mosquito infectivity [35]). To illustrate the epidemiological importance of vertebrate hosts with relatively low gametocytaemia, all additional graphs have a logged x-axis. Figures 2B and 2E show how TBIs targeting different life-stages will influence the final presence and density of salivary gland sporozoites within the mosquito. Precisely which parasite life-stage is best to target to achieve the greatest reduction in transmission will depend on intervention efficacy, number of gametocytes ingested, and on whether malaria transmission intensity is assumed to depend on the presence or the density of salivary gland sporozoites (Figures 2C and 2F). Targeting the ookinete life-stage is the most successful strategy when the intervention is highly efficacious or the mosquito ingests a low/intermediate number of parasites (Figure 2C). This is because the Allee-effect (positive density dependence) acts on the production of oocysts at low ookinete densities (Figure 1). Reducing ookinete density per mosquito beneath a certain threshold (212 in this model system) would enhance parasite control by making the development of ookinetes to oocysts less efficient, giving additional benefits to TBIs. An intervention reducing ookinete density by 60% (blue line) in mosquitoes ingesting less than 100,000 macrogametocytes has a greater than 60% reduction in the density of salivary gland sporozoites (Figure 2E).

### Parasite life-stage causing vector mortality

The results of the above model were used to re-fit the relationship between parasite density and mosquito mortality rate using the same data and methodology presented in [7] (see Additional file 1). The model that used the number of salivary gland sporozoites as the explanatory variable gave the best fit to the experimental data (AIC = -3384), followed by oocysts (AIC = -3376) and finally ookinetes (AIC = -3370). However, this result should be interpreted with caution. Though the AIC values were able to clearly distinguish between the models that used sporozoites or ookinetes as the explanatory variable, the difference between the sporozoite and oocyst models was not so great as to exclude either model. The best-fit models were used to show the relationship between the number of gametocytes ingested and the number of infectious bites made by a mosquito during its lifetime (see Additional file 2).

The complex relationship between the aggregated parasite distribution and the range of efficacies generated by a potential TBV makes the impact of parasite-induced vector mortality far from intuitive. Ultimately, the impact of parasite-induced vector mortality will depend on the time it takes for an infected mosquito to become infectious, the shape of the relationship between parasite density and mosquito mortality and the distribution of parasites within the population (both before and after an intervention). If vector mortality is determined by the number of oocysts, a mosquito that ingests 100,000 macrogametocytes will bite, on average, 21% fewer times from 16 days after blood-feeding onwards (i.e., once it is infectious, see Additional file 1) than an uninfected mosquito (a drop from 3.8 to 3.0). This figure drops to 13% (from 3.8 to 3.3) if vector mortality is caused by the number of sporozoites within the salivary glands.

### Overall transmission prior to intervention

**Figure 3:**
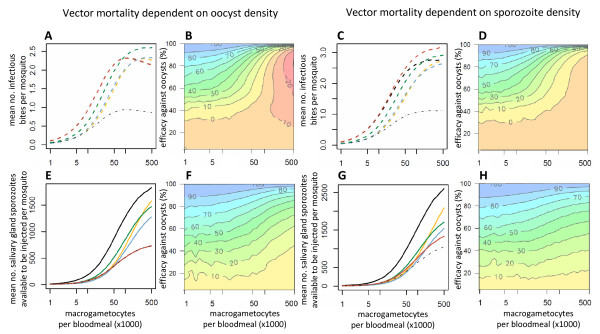
**The impact on transmission of interventions which target different parasite life-stages**. The Figure shows the relationship between the number of macrogametocytes ingested and transmission according to whether the latter is dependent on the presence (dashed lines, panels A, B, C and D) or density (solid lines, panels E, F, G and H) of salivary gland sporozoites. The mean number of infectious bites per mosquito and the lifetime mean number of salivary gland sporozoites available to be injected per mosquito are both estimated assuming the mosquito is infected at its first bloodmeal. Parasite-induced vector mortality is dependent on the number of oocysts at day 10 (A, B, E and F) or salivary gland sporozoites on day 21 (C, D, G and H). In panels A, C, E and G the model was run with either no intervention (black line) or representing an intervention which reduced the production of a life-stage by 60%: gametocytes (yellow line); ookinetes (blue line); oocysts (green line); salivary gland sporozoites (red line). Note that the black and red lines overrun each other in panel A. The thin grey dotted-dashed line indicates an overall efficacy of 60% for comparison. The contour plots (panels B, D, F and H) show the relationship between gametocytaemia, the efficacy of an intervention which targeting oocysts and the overall reduction in transmission that this intervention causes.

The cumulative impact of density-dependent sporogonic development and vector mortality on overall transmission is shown in Figure 3. If the intensity of malaria transmission were determined by parasite density, then the reduction in the number of infectious bites at high parasite densities would be offset by a net increase in the number of sporozoites reaching the salivary glands. Therefore, in this scenario the highest contribution to transmission would be made by mosquitoes that ingest the greatest number of gametocytes (Figures 3E and 3G). However, if malaria transmission were determined solely by the number of infectious bites, then increases in parasite density above a certain threshold would have only a minimal impact on mosquito infectivity. Mosquitoes that feed on hosts with intermediate gametocytaemia densities could therefore make the highest contribution to overall transmission. This is seen in the absence of an intervention in Figure 3A, in which transmission peaks in mosquitoes that ingest ~100,000 macrogametocytes per bloodmeal, if vector mortality is determined by oocyst density. However, if vector mortality were determined by sporozoite density (Figure 3C), then the small increase in the prevalence of infectious mosquitoes as the number of macrogametocytes rises above 200,000 per bloodmeal is enough to counteract the reduction in the mean number of infectious bites made by the mosquito population during their lifetime. This is in part because the highly aggregated distribution of parasites within the vector population ensures that a high percentage of the parasites are within heavily infected mosquitoes, which will not survive long enough to contribute to transmission. Re-running the model assuming that parasites are Poisson-distributed within the mosquito population increases the modal relationship between gametocytaemia and transmission (see Additional file 3).

### Overall transmission after an intervention

A TBI with low efficacy could elevate transmission intensity above that seen prior to the intervention. For example, if parasite-induced vector mortality were determined by oocyst density in the mosquito, then according to the model, the maximal average number of infectious bites during the lifetime of a mosquito prior to an intervention would be 2.3. This could increase to 2.9 if a TBI reducing oocyst density by approximately 60% was introduced (Figure 3A). This is because mosquitoes that ingest a high number of gametocytes have a greater chance of becoming infected, but a lower life expectancy. If an intervention reduced the number of parasites of the life-stage that causes vector mortality, the life-expectancy of the mosquito would increase, boosting transmission.

Again, the impact of TBIs that target different sporogonic stages on the overall level of transmission will depend on the efficacy of the intervention, the number of gametocytes ingested by the mosquito and the biology of the infection (Figure 3). The likelihood of an intervention increasing transmission will depend on which parasite life-stage the intervention is targeting and which life-stage vector mortality is dependent on. Interventions are less likely to have a negative effect in mosquitoes which ingest a low number of gametocytes. However, if vector mortality is caused by later sporogonic stages then interventions with low efficacies which target oocysts or sporozoites may have a perverse outcome irrespective of how many gametocytes the mosquito ingests, though the size of the effect is likely to be modest (Figures 3B and 3D).

**Figure 4:**
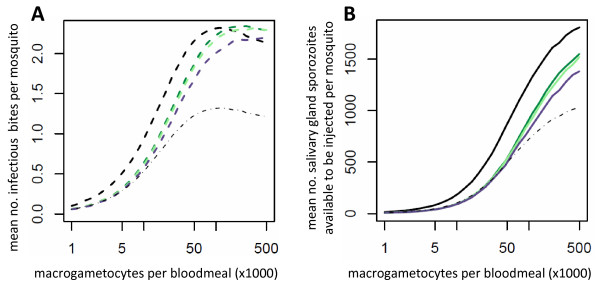
**The relationship between gametocytaemia and transmission for three different theoretical transmission blocking vaccines which have the same mean efficacy but differ in their variance**. The two panels show the impact of the interventions which reduce the production of ookinetes by an average of 60% if malaria transmission is dependent on the presence (dashed line, panel A) or density (solid line, pane B) of salivary gland sporozoites. Predictions are shown for no intervention (black line) or for a TBV which generates a range of antibody responses in the vaccinated population corresponding to a ratio of the 97.5% percentile to the 2.5% percentile of 9 fold (low variance, light green), 68 fold (medium variance, dark green) or 6664 fold (high variance, purple) [31].

Parasite-induced vector mortality will make the effectiveness of an intervention depend on its ability to reduce parasite prevalence as well as parasite density. A partially effective intervention with a high variance in efficacy will tend to cause a greater reduction in transmission than one with a lower variance, as it will have a the greatest chance of clearing infection in some mosquitoes whilst minimising the number of bites made by those still contributing to transmission (Figure 4).

## Discussion

Density-dependent processes regulating malaria parasite development within the mosquito are likely to influence the success of partially effective TBIs and should be considered when making decisions on which stage(s) to prioritize as target(s). The complex relationships between different positive and negative density-dependent processes mean that the life-stage that is best to target may also vary between settings. However, some general conclusions can be drawn from this analysis. Interventions that reduce ookinete density beneath a threshold are likely to have auxiliary benefits as they utilize positive density-dependent processes naturally restricting sporogonic development at low densities. TBIs which fail to reduce ookinete density beneath this threshold will have a lower overall impact on transmission than the efficacy achieved at the targeted life-stage. Unless the intervention successfully clears the infection, any reductions in parasite density could potentially increase the life-expectancy of the mosquito, enhancing transmission. Therefore partially effective interventions with a high variance in effectiveness will be more effective than those that have a more uniform efficacy.

Model systems provide a powerful tool for identifying testable hypotheses that may be applicable to the transmission of human malaria. The processes investigated in this paper have been identified and quantified in the *P. berghei-An. stephensi *laboratory model and this work needs to be repeated in naturally found parasite-vector combinations to draw definitive conclusions about human malaria. Similar density-dependent processes influencing sporogonic development within the mosquito have been identified in other parasite-vector combinations [8,36]. Indeed, a number of studies of *P. falciparum *indicate a sigmoidal relationship between gametocyte density and mosquito infectivity [10-14], indicating that positive and negative regulatory processes within the mosquito may influence the population dynamics of human malaria.

This paper has examined the effect of different potential TBIs on mosquitoes fed on blood with known gametocyte concentrations. More accurate estimation of an intervention's impact at the population level will require information on the distribution of gametocytes within the bitten human population, and this is likely to vary temporally [37], geographically [14], and with treatment [38]. As a result, the community impact of different TBIs will likely vary from setting to setting. Gametocyte densities of *Plasmodium falciparum *are lower than those in the experimental system investigated here, with a significant proportion of human hosts having densities too low to detect through standard microscopy [34]. This may improve the efficacy of TBIs if ookinete densities were low enough to restrict transmission as a consequence of the positive density-dependent processes that may operate on oocyst development.

The chance that a TBI actually increases the average number of infectious bites a human host population is exposed to will depend on the severity of parasite-induced vector mortality, the distribution of parasites within the human host and mosquito population before and after the intervention, and the shape of the relationship between gametocyte density and mosquito infectivity. The probability of infecting a feeding mosquito plateaus at moderate gametocyte densities, though the exact shape of this gametocytaemia-infectivity relationship may vary [39-41]. Therefore, partially decreasing gametocyte density in human hosts with high gametocytaemia may not substantially decrease the host's ability to infect a mosquito. In such a scenario, even weak density-dependent parasite-induced vector mortality would reduce the number of infectious bites made by vectors ingesting a high number of gametocytes. The consequences of this for malaria transmission will depend on the distribution of gametocytes within the human host population. An intervention may reduce the contribution to transmission of hosts with low gametocytaemia whilst at the same time increasing the number of infectious bites made by mosquitoes (during their lifetime) that feed on highly gametocytaemic hosts. The overall change in the potential for malaria transmission will therefore depend on whether the cumulative reduction in transmission from human hosts with low number of gametocytes outweighs the increase in transmission caused by heavily infected hosts.

The model has been parameterized using mosquito mortality data from laboratory experiments where the insects will live much longer than they would do under natural conditions. In the wild, mosquitoes are likely to make relatively few bites more than 10 days after becoming infected, so malaria transmission will be highly sensitive to small changes in mosquito mortality. In addition, older mosquitoes are much more susceptible to changes in parasite density [7]. Since these are the mosquitoes contributing most to transmission (given the long extrinsic incubation period of *Plasmodium *within *Anopheles*) even a small increase in mortality of this age group may have a significant impact on transmission.

The results of this analysis indicate that interventions with an intermediate or low efficacy are more likely to cause an increase in transmission. Although it is unlikely that any TBI with a low efficacy would be deployed in a public health programme, the effectiveness of an intervention may wane over time so it is important to consider how these low efficacies will influence transmission dynamics. For example, TBV efficacy may decline relatively rapidly as the concentration of antibodies within the bloodstream falls. This means that unless vaccine efficacy is regularly boosted, antibody concentration may drop, causing the level of transmission to increase above pre-intervention levels. This is in additions to any enhancement of mosquito infectivity that may occur as the concentration of serum antibodies falls [42-44].

It is interesting to note that if parasite-induced vector mortality operates in natural combinations of medical importance, then *Plasmodium *may benefit from eliciting a mild transmission-blocking immune response. If malaria transmission is determined by sporozoite presence (rather than density), then reducing parasite density once the mosquito is infected will tend to increase overall transmission by reducing the mortality of the insect vector. Pre-fertilization antigens are known to be a target of the natural immune response and it may be more plausible to ascribe this type of immunity to an evolutionary response by the parasite and not the human host.

Further work is required to identify whether parasite-induced vector mortality acts in malaria endemic regions so that any perverse outcome of TBIs can be thoroughly understood and contained. Taking into account the possible non-linear relationships between different sporogonic life-stages will increase the chance of identifying any changes in mosquito mortality caused by the parasite. Re-fitting vector mortality data indicates that later sporogonic stages are the most likely cause of parasite-induced vector mortality. However, it is likely that more than one parasite life-stage will play a role, as oocysts or sporozoites cannot have caused the elevated mortality seen in mosquitoes immediately after blood feeding.

Research into possible TBIs should consider how the development of the parasite within the mosquito may influence study results. The standard method of assessing the efficacy of a TBV is to compare the number of oocysts within mosquitoes fed on immunised/unimmunised blood [45,46]. These efficacy estimates therefore already include the net effects of all density-dependent mechanisms acting upon the production of ookinetes and oocysts. Interventions targeting gametocytes and ookinetes may have had a different efficacy than that measured at the oocyst stage, depending on the number of gametocytes ingested. Understanding this may improve the accuracy of molecular methods for assessing TBV efficacy [46]. Researchers should also be aware that reductions in oocyst intensity may overestimate reductions in malaria transmission due to the actions of density-dependent sporozoite development and parasite-induced vector mortality.

In light of the possible impact of parasite-induced vector mortality, lab-based studies measuring intervention efficacy should compare the survival of mosquitoes between treatment groups over their whole lifetime. This is because vectors that ingest a lower number of parasites may have had a higher probability of surviving long enough to be dissected. Failing to control for this may cause the intervention efficacy to be overestimated. It is also important to investigate the impact of an intervention on the survival of older mosquitoes as they make the greatest contribution to overall transmission. Studies should quantify the distribution of efficacies caused by a TBI (both within the human host and the mosquito) to allow the full impact of a control measure to be estimated. It is also important to understand how the efficacy of an intervention may change over time. Phase II clinical trials of possible TBV candidates should quantify the rate and variance of antibody decay in a range of environmental settings (i.e. with different degrees of natural immunological boosting from ongoing infection). In addition, ideally the duration of both Phase II and Phase III trials should be lengthened until target antibody concentrations have returned to pre-intervention levels, allowing the full impact of starting an intervention but failing to sustain it to be quantified.

There is evidence that other regulatory processes acting upon the malaria parasite within the mosquito may also be density-dependent in natural populations. For example, *Plasmodium *may influence mosquito biting frequency [47], feeding persistence [48] and physiology [49]. Other factors such as parasite sex ratio may also affect the likelihood of onwards transmission [50], and therefore should be considered when quantifying the overall impact of an intervention. Similar methods should be used to understand the influence of multiple density-dependent processes on other vector-borne diseases as parasite development and vector survival have been shown to be influenced by the parasite density in both human onchocerciasis [51,52] and lymphatic filariasis [53-55].

## Conclusions

This paper has highlighted the importance of understanding the population dynamics of the malaria parasite within the mosquito as processes regulating the development of the parasite may enhance or impede the effectiveness of a control strategy. Mathematical models should be used to investigate how an intervention influences both parasite development and vector mortality in order to fully evaluate the effectiveness of the new transmission blocking interventions currently under development.

## List of abbreviations

TBI: (Transmission-blocking interventions); TBV: (Transmission-blocking vaccine).

## Competing interests

The authors declare that they have no competing interests.

## Authors' contributions

TSC carried out the mathematical modelling and drafted the manuscript. TSC, EJD and MGB conceived the study. EJD, RES, GKC, JCK and MGB participated in the design of the study and helped to draft the manuscript. All authors read and approved the final manuscript.

## Supplementary Material

Additional file 1 **Description of the mathematical model and table of parameter values**. Click here for file

Additional file 2 **The impact of varying which parasite life-stage causes mosquito mortality on the mean number of potentially infectious bites made by a mosquito in its lifetime**. Parasite mortality is dependent on the mean number of ingested ookinetes (blue line), oocysts at day 10 (green line) or salivary gland sporozoites on day 21 (red line). All bites made 10 days after the first bloodmeal are defined as being potentially infectious. The relationship between ookinete density and the later life-stages was estimated using the full model and used to re-fit the mosquito mortality data from [7]. Click here for file

Additional file 3 **The relationship between gametocytaemia and transmission for a Poisson-distributed parasite population**. The potential for malaria transmission is dependent on either salivary gland sporozoite density (solid lines, panel A) or prevalence (dashed lines, panel B). The mean number of salivary gland sporozoites available to be injected per mosquito and the mean number of infectious bites per mosquito are both estimated assuming the mosquito is infected at its first bloodmeal and correspond to the total during the mosquito's lifetime. The model was run with either no intervention (black line) or representing a TBI which reduced the production of a life-stage by 60%: gametocytes (yellow line); ookinetes (blue line); oocysts (green line); salivary gland sporozoites (red line). The thin dotted-dashed grey line (which overlaps with the red line in A) indicates an overall reduction in sporozoite density/prevalence efficacy of 60% as a benchmark for comparison. The number of parasites of each life-stage among the mosquitoes follows a Poisson distribution (i.e. $k_{i}^{j}$ = 10). Click here for file
