# Supplementary material for: Population biology of malaria within the mosquito: density-dependent processes and potential implications for transmission-blocking interventions
Source: Malar J. 2010 Nov 4;9:311. doi: 10.1186/1475-2875-9-311 (PMC2988043; doi:10.1186/1475-2875-9-311)
Supplement: Additional file 1 — Description of the mathematical model and table of parameter values. [file 1475-2875-9-311-S1.DOC]

**Description of model structure**

The model tracks the number of *Plasmodium* parasites of each life-stage at different times after blood-feeding as described by Sinden *et al.* [2] (i.e. the number of macrogametocytes ingested, ookinetes 15hours after feeding, oocysts at day 10 and sporozoites in the salivary glands at day 21). Let $L_{i}^{j}$indicate the number of parasites of life-stage$j$within mosquito $i$, be it macrogametoctyes ($L_{i}^{1}$), ookinetes ($L_{i}^{2}$), oocysts ($L_{i}^{3}$) or sporozoites in the salivary glands ($L_{i}^{4}$).The efficacy of a transmission-blocking intervention (TBI) against the targeted life-stage is given by parameter $E_{i}^{j}$which denotes the percentage reduction in the number of parasites reaching life-stage $j$ within mosquito $i$, caused by an intervention targeting that life-stage. To aid clarity we assume that each intervention only affects the production of a specific life-stage and its efficacy does not depend on parasite density within the host or mosquito [46]. For example, a transmission-blocking vaccine (TBV) which kills 90% of ookinetes as they are produced but has no impact on the other sporogonic stages would have the following efficacy values $E_{i}^{1}=E_{i}^{3}=E_{i}^{4}=0$,$E_{i}^{2}=90$. For comparability it is assumed that interventions reduce the production of the specific life-stage at which they are targeted, and not the production of a subsequent stage. This is analogous to TBV antibodies attacking surface proteins of a particular life-stage as soon as they appear. The epidemiological importance of upon which life-stage a TBI operates will depend on which life-stage is causing parasite-induced vector mortality. For example, if vector mortality is dependent on the numbers of ookinetes penetrating the mosquito midgut, then it will make very little epidemiological difference whether a TBV kills ookinetes as they develop or inhibits their penetration of the midgut epithelium. However, if parasite-induced vector mortality is dependent on the number of sporozoites entering the salivary glands, then transgenic mosquitoes in which the survival of sporozoites already established is reduced (e.g. through the over-expression of parasite antagonists) may have a different impact on transmission from those by mosquitoes in which it is the penetration of the salivary gland epithelia that is impeded.

Human populations tend to generate a log-normal distribution of antibody concentrations when vaccinated [30]. If it is assumed that mosquitoes bite vaccinated hosts at random then the range of intervention efficacies for a TBV within the mosquito population can be described by the Hill equation,

$E_{i}^{j}=\frac{Ab_{i}^{1.68}}{\left(1+Ab_{i}^{1.68}\right)}$ [1]

where $Ab_{i}$ is the antibody concentration within mosquito $i$, which has a log normal distribution (whose ratio of upper to lower 95% limit is nine fold, see [31]). A nine fold range in antibody response was chosen from the values presented by [31] as this was the value which best fitted the prevalence / intensity efficacy relationship measured by [46] (also see Figure S2 for a graph representing a range of antibody concentration variances). The number of macrogametoctes fed to mosquito $i$at time 0 is denoted $l_{i}^{1}$. The number of ookinetes, oocysts and sporozoites which develop in mosquito $i$ in the absence of an intervention, $l_{i}^{j}$, is randomly selected from a negative binomial distribution using the following equation,

$l_{i}^{j}\sim\text{NegBin}\left[y_{i}^{j}\left(L_{i}^{j-1}\right),k_{i}^{j}\left(y_{i}^{j}\right)\right]$ [2]

$k_{i}^{j}\left(y_{i}^{j}\right)=\kappa_{w}^{j}+\frac{\kappa_{x}^{j}\left(y_{i}^{j}\right)^{\kappa_{y}^{j}}}{1+\kappa_{z}^{j}\left(y_{i}^{j}\right)^{\kappa_{y}^{j}}}$ [3]

where $j$ is the life-stage under investigation, $k_{i}^{j}\left(y_{i}^{j}\right)$is the overdispersion parameter of the negative binomial distribution of parasite life-stages among mosquitoes, which itself depends on parasite density as described in [2]. The overdispersion parameter is an inverse function of the degree of parasite aggregation such that very small values correspond to a highly overdispersed distribution and values greater than 5 correspond to a random, Poisson distribution. The function $y_{i}^{j}\left(L_{i}^{j-1}\right)$relates the mean number of parasites in life-stage$j$ to the number in the previous life-stage, $L_{i}^{j-1}$, using the following equations [2],

$y_{i}^{j}\left(L_{i}^{j-1}\right)=\frac{\alpha^{j}\left(L_{i}^{j-1}\right)^{\beta^{j}}}{1+\gamma^{j}\left(L_{i}^{j-1}\right)^{\beta^{j}}}$. [4]

Therefore the number of macrogametoctyes ($L_{i}^{1}$), ookinetes ($L_{i}^{2}$), oocysts ($L_{i}^{3}$) or sporozoites ($L_{i}^{4}$) after the operation of density dependence and the reduction exerted by the TBI is given by,

$L_{i}^{j}=l_{i}^{j}\left(100-E_{i}^{j}\right)/100$. [5]

A full list of parameter definitions and values (and their 95% confidence intervals) are given in Table S1. Graphical representation of the model is given Figure 1 of the main text. The ookinete (as a function of macrogametocytes) and sporozoite (as a function of oocysts) density-dependent functions are hyperbolic curves which represent a limitation in population size as the numbers of parasites of the previous stage increase. The function determining the number of oocysts produced from ookinetes is a sigmoidal curve representing a process by which increasing parasite numbers initially facilitates but later restricts population size. The parameter representing the degree of parasite overdispersion in the distribution of each of these sporogonic stages among mosquitoes is also density dependent (as described by equation [30]), with parameters given in Table S1 [2]. The transition from oocyst to sporozoites cannot be measured directly as mosquitoes are fatally damaged during the oocyst counting procedure. In the report by Sinden *et al*. [2], the number of sporozoites produced by each oocyst was estimated by feeding mosquitoes a known concentration of ookinetes, counting the number of observed oocysts, and predicting the expected number of oocysts in each mosquito using$y_{i}^{3}\left(L_{i}^{2}\right)$. To prevent mosquitoes which did not develop oocysts from influencing the distribution of sporozoites, the best fit model of $k_{i}^{3}\left(y_{i}^{3}\right)$ was re-fitted from the original Sinden *et al.* 2007 data assuming that all oocyst-positive mosquitoes produce sporozoites.

**Parasite life-stage causing vector mortality.** The survival function of a mosquito at time $t$ after the cessation of first engorgement is described (from [7] by,

$\lambda_{i}\left(t,L_{i}^{j}\right)=\exp\left\{-\left[\left(\nu_{0}+\nu_{1} L_{i}^{j}\right)\frac{t^{3}}{3}-\left(\delta_{0}+\delta_{1} L_{i}^{j}\right)\frac{t^{2}}{2}+\left(\theta_{0}+\theta_{1} L_{i}^{j}\right)\right]\right\}$ [6]

where $j$ denotes which parasite life-stage influences mosquito survival and the expression within the square brackets is the parabolic hazard that best fitted the instantaneous mosquito mortality rate data as a function of time post-feeding and parasite density of Dawes et al. [7]. Parameter values were estimated by re-fitting the hazard contained in equation [6] to the original mortality data from [7] using each parasite life-stage in turn and comparing with the results with those presented in [7], where it had been assumed that parasite-induced vector mortality is caused by the ookinete stage. The resulting fits were compared via the Akaike information criterion (AIC). When comparing two models, a difference greater than 10 between the respective AICs is considered as empirical evidence in favor of the model with the lower AIC value [29]. The individual-based model presented above was used to fit the relationship between ookinetes and sporozoites as combining multiple non-linear functions would have overestimated the number of sporozoites at high ookinete densities. Integrating $\lambda_{i}\left(t,L_{i}^{j}\right)$with respect to time gives the life-expectancy of mosquito $i$at cessation of engorgement, $\Lambda_{i}\left(L_{i}^{j}\right)$.

For simplicity it is assumed that all sporozoites are within the salivary glands $n$ days after feeding. If mosquitoes bite once every $1/a$ days then the number of potentially infectious bites made in the lifetime of mosquito $i$,$\Phi_{i}\left(L_{i}^{j}\right)$, is given by,

$\Phi_{i}\left(L_{i}^{j}\right)=\left\{\begin{matrix} 0 \Lambda_{i}\left(L_{i}^{j}\right)<n \\ \left[\Lambda_{i}\left(L_{i}^{j}\right)-n\right]a \Lambda_{i}\left(L_{i}^{j}\right)\geq n \end{matrix}\right.$. [7]

**Prevalence of infectious mosquitoes.** If $S_{i}$denotes whether mosquito $i$has at least one sporozoite (i.e. $S_{i}$ = 1 if $L_{i}^{4}\geq1$ and 0 otherwise) then the mean number of infectious bites made by the mosquito population over the lifetime of a mosquito infected during its first bloodmeal, denoted by $T^{P}$, is given by,

$T^{P}=\sum_{i=1}^{r} \Phi_{i}\left(L_{i}^{j}\right)S_{i}/r$ [8]

where $r$ is the number of mosquitoes in the population. For simplicity the model assumes that the number of sporozoites injected by a mosquito during a bite does not significantly reduce the number of salivary gland sporozoites.

**Density of salivary gland sporozoites.** Alternatively, the mean number of salivary gland sporozoites available to be injected over the lifetime of a mosquito infected during its first bloodmeal, $T^{I}$, is

$T^{I}=\sum_{i=1}^{r} \Phi_{i}\left(L_{i}^{j}\right)L_{i}^{4}/r$. [9]

Confidence interval estimates were generated by re-running the model to get the lowest or highest possible transmission using the confidence interval estimates given in Table S1.

**Table 1. Parameter values and definitions.** Values are taken from [2] & [7] unless otherwise stated

| Symbol | Description | Values (and 95% CI) |
| --- | --- | --- |
| $j$ | parasite life-stage under investigation | macrogametocytes ($j=1$)  ookinetes ($j=2$)  oocysts ($j=3$)  salivary gland sporozoites ($j=4$) |
| $L_{i}^{j}$ | number of parasites of life-stage $j$ within mosquito $i$after density dependence and intervention | equation [5] |
| $l_{i}^{j}$ | number of parasites of life-stage $j$within mosquito $i$ in the absence of control interventions | equation [2] |
| $E_{i}^{j}$ | percentage efficacy of a transmission-blocking intervention against life-stage $j$ within mosquito $i$ | equation [1] |
| $y_{i}^{2}\left(L_{i}^{1}\right)$ | function describing the mean number of ookinetes developing within mosquito $i$from ingested gametocytes (equation [4], hyperbolic function; *β* = 1) | $\alpha^{2}=\text{0.013 }\left(0.012,0.014\right)$ $\gamma^{2}=3.6\times10^{\text{-6}}\left(1.1\times10^{\text{-6}},4.7\times10^{\text{-6}}\right)$ |
| $y_{i}^{3}\left(L_{i}^{2}\right)$ | function describing the mean number of oocysts developing within mosquito $i$from ookinetes (equation [4], sigmoidal function,* β* > 1) ^‡^ | $\alpha^{3}=5.8\times10^{\text{-4}} \left(5.6\times10^{\text{-4}},6.1\times10^{\text{-4}}\right)$ $\gamma^{3}=7.4\times10^{\text{-6}} \left(6.9\times10^{\text{-6}},8.1\times10^{\text{-6}}\right)$ $\beta^{3}=1.74 \left(1.73,1.76\right)$ |
| $y_{i}^{4}\left(L_{i}^{3}\right)$ | function describing the mean number of salivary gland sporozoites developing within mosquito $i$ from oocysts (equation [4], hyperbolic function) | $\alpha^{4}=\text{62.21 }\left(53.98,72.20\right)$ $\gamma^{4}=0.018 \left(0.013,0.024\right)$ |
| $k_{i}^{2}\left(y_{i}^{2}\right)$ | distribution of ookinetes within the mosquito population (equation [3], hyperbolic function, *κ_w_* = 0; *κ_y_* = 1) | $\kappa_{x}^{2}=6.5\times10^{\text{-3}} \left(5.1\times10^{\text{-3}},7.9\times10^{\text{-3}}\right)$ $\kappa_{z}^{2}=1.6\times10^{\text{-3}} \left(1.2\times10^{\text{-3}},2.4\times10^{\text{-3}}\right)$ |
| $k_{i}^{3}\left(y_{i}^{3}\right)$ | distribution of oocysts within the mosquito population (equation [3], power function, *κ_z_* = 0) | $\kappa_{w}^{3}= 0.54\left(0.\text{43,0}.64\right)$ $\kappa_{x}^{3}=3.1\times10^{\text{-4}} \left(2.5\times10^{\text{-4}},3.6\times10^{\text{-4}}\right)$ $\kappa_{y}^{3}= 1.81\left(1.77\text{,1.84}\right)$ |
|  | distribution of salivary gland sporozoites within the mosquito population (equation [3], linear function with zero intercept, *κ_w_* = *κ_z_* = 0; *κ_y_* = 1) | $ |
| $\lambda_{i}\left(t,L_{i}^{2}\right)$ | survivorship function of mosquito $i$ at time $t$. Superscript$j=2$ denotes that vector mortality depends on ookinete density (see equation [6])^‡^ | $\nu_{0}^{4}=1.49\times10^{-4}$;$\nu_{1}^{4}=3.81\times10^{-8}$ $\delta_{0}^{4}=4.13\times10^{-3}$;$\delta_{1}^{4}=7.72\times10^{-7}$ $\theta_{0}^{4}=3.90\times10^{-2}$;$\theta_{1}^{4}=6.32\times10^{-6}$ |
| $\lambda_{i}\left(t,L_{i}^{3}\right)$ | survivorship function of mosquito $i$ at time $t$. Superscript$j=3$ denotes that vector mortality depends on oocyst density (see equation [6]) | $\nu_{0}^{3}=1.54\times10^{-4}$;$\nu_{1}^{3}=4.47\times10^{-6}$ $\delta_{0}^{3}=4.24\times10^{-3}$;$\delta_{1}^{3}=9.22\times10^{-5}$ $\theta_{0}^{3}=4.03\times10^{-2}$;$\theta_{1}^{3}=16.74\times10^{-4}$ |
| $\lambda_{i}\left(t,L_{i}^{4}\right)$ | survivorship function of mosquito $i$ at time $t$. Superscript$j=4$ denotes that vector mortality depends on sporozoite density (see equation [6]) | $\nu_{0}^{4}=1.50\times10^{-4}$;$\nu_{1}^{4}=7.28\times10^{-8}$ $\delta_{0}^{4}=4.12\times10^{-3}$;$\delta_{1}^{4}=1.58\times10^{-6}$ $\theta_{0}^{4}=3.98\times10^{-2}$;$\theta_{1}^{4}=1.02\times10^{-5}$ |
| $\Lambda_{i}\left(L_{i}^{j}\right)$ | life expectancy of mosquito $i$ after the cessation of engorgement | $\Lambda_{i}\left(L_{i}^{j}\right)=\int_{t=0}^{\infty} \lambda_{i}\left(t,L_{i}^{j}\right)dt$ |
| $a$ | biting rate per mosquito on humans | 0.3 day^–1*^ |
| $n$ | mean duration of the extrinsic incubation period within mosquito | 16 days^†^ |
| $r$ | number of mosquitoes in the population | 100,000 |
| $\Phi_{i}$ | number of potentially infectious bites made by mosquito $i$ during its lifetime | equation [7] |
| $T^{P}$ | mean number of infectious bites made by the mosquito population over the lifetime of a mosquito infected during its first bloodmeal |  |
| $T^{I}$ | mean number of salivary gland sporozoites available to be injected over the lifetime of a mosquito infected during its first bloodmeal |  |

^‡^ adjusted to make the relationship dependent on the number of ookinetes per mosquito and not ookinetes per µl blood (assuming an average bloodmeal volume of 2.13 µl as in [2])

^$^ mosquitoes were fed with ookinetes so the exact number of oocysts in each vector is unknown and was estimated using. To prevent mosquitoes which did not develop oocysts from influencing the distribution of sporozoites the best fit model of was refit from the original data [2], using the same methodology but excluding all zero counts (see text).

^*^ from [33]

^†^ unpublished results for the mean latent period of the parasite-vector combination at 19 ⁰C (the temperature studies [2] and [7] were conducted at)
