# Supplementary material for: Population biology of malaria within the mosquito: density-dependent processes and potential implications for transmission-blocking interventions
Source: Malar J. 2010 Nov 4;9:311. doi: 10.1186/1475-2875-9-311 (PMC2988043; doi:10.1186/1475-2875-9-311)
Supplement: Additional file 3 — The relationship between gametocytaemia and transmission for a Poisson-distributed parasite population. The potential for malaria transmission is dependent on either salivary gland sporozoite density (solid lines, panel A) or prevalence (dashed lines, panel B). The mean number of salivary gland sporozoites available to be injected per mosquito and the mean number of infectious bites per mosquito are both estimated assuming the mosquito is infected at its first bloodmeal and correspond to the total during the mosquito's lifetime. The model was run with either no intervention (black line) or representing a TBI which reduced the production of a life-stage by 60%: gametocytes (yellow line); ookinetes (blue line); oocysts (green line); salivary gland sporozoites (red line). The thin dotted-dashed grey line (which overlaps with the red line in A) indicates an overall reduction in sporozoite density/prevalence efficacy of 60% as a benchmark for comparison. The number of parasites of each life-stage among the mosquitoes follows a Poisson distribution (i.e. kij = 10). [file 1475-2875-9-311-S3.DOC]

B

A
