# Supplementary material for: Population biology of malaria within the mosquito: density-dependent processes and potential implications for transmission-blocking interventions
Source: Malar J. 2010 Nov 4;9:311. doi: 10.1186/1475-2875-9-311 (PMC2988043; doi:10.1186/1475-2875-9-311)
Supplement: Supplementary file 3 [file 1475-2875-9-311-S2.DOC]

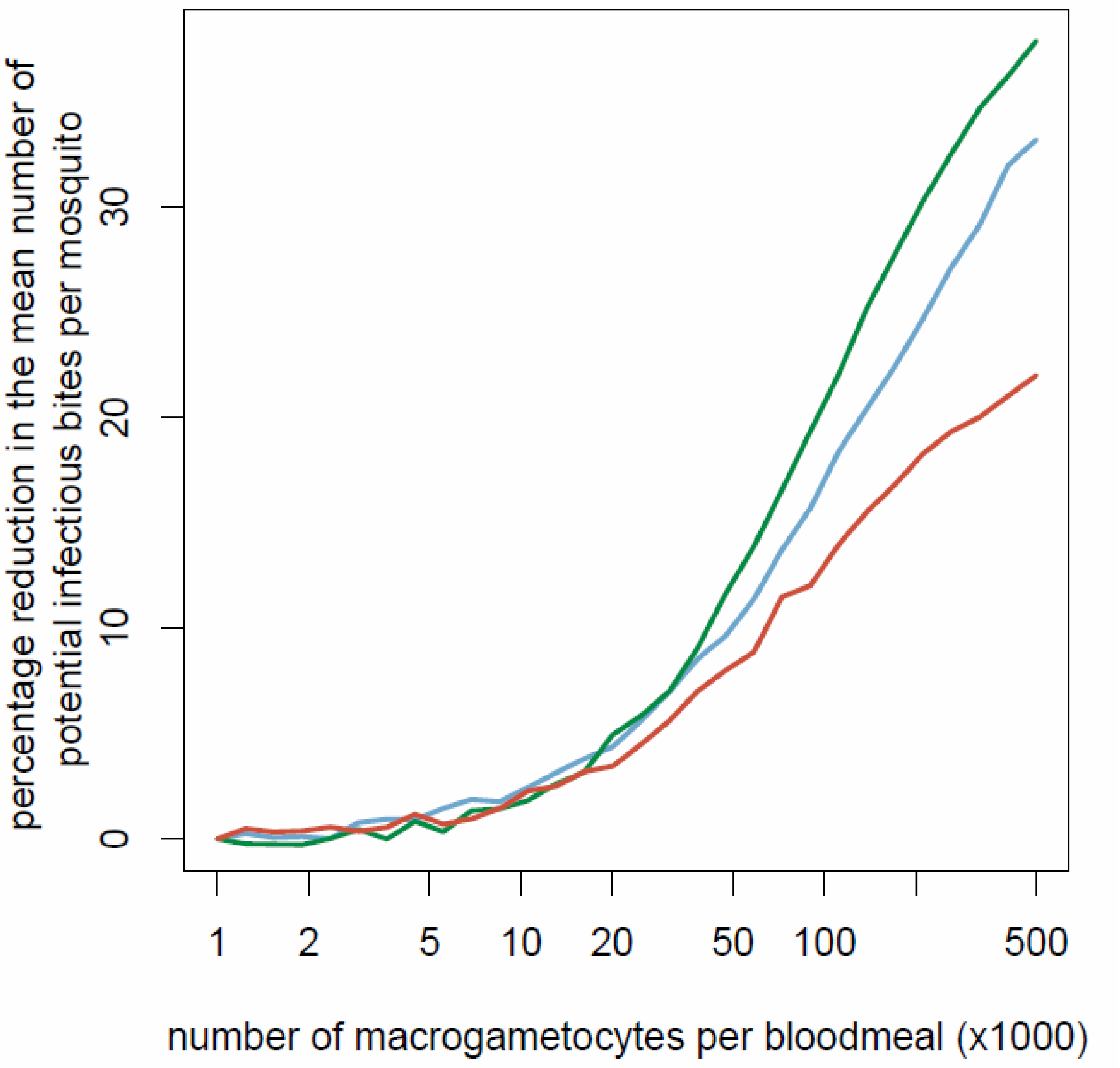
percentage reduction in the mean number of potential infectious bites per mosquito
0
10
20
30
1
2
5
10
20
50
100
500
number of macrogametocytes per bloodmeal (x1000)
